# Developing Ethics and Equity Principles, Terms, and Engagement Tools to Advance Health Equity and Researcher Diversity in AI and Machine Learning: Modified Delphi Approach

**DOI:** 10.2196/52888

**Published:** 2023-12-06

**Authors:** Rachele Hendricks-Sturrup, Malaika Simmons, Shilo Anders, Kammarauche Aneni, Ellen Wright Clayton, Joseph Coco, Benjamin Collins, Elizabeth Heitman, Sajid Hussain, Karuna Joshi, Josh Lemieux, Laurie Lovett Novak, Daniel J Rubin, Anil Shanker, Talitha Washington, Gabriella Waters, Joyce Webb Harris, Rui Yin, Teresa Wagner, Zhijun Yin, Bradley Malin

**Affiliations:** 1 National Alliance Against Disparities in Patient Health Woodbridge, VA United States; 2 Vanderbilt University Medical Center Nashville, TN United States; 3 Yale University New Haven, CT United States; 4 University of Texas Southwestern Medical Center Dallas, TX United States; 5 Fisk University Nashville, TN United States; 6 University of Maryland, Baltimore County Baltimore, MD United States; 7 OCHIN Portland, OR United States; 8 Temple University Philadelphia, PA United States; 9 Meharry Medical College Nashville, TN United States; 10 AUC Data Science Initiative Clark Atlanta University Atlanta, GA United States; 11 Morgan State University Center for Equitable AI & Machine Learning Systems Baltimore, MD United States; 12 University of Florida Gainesville, FL United States; 13 University of North Texas Health Science Center SaferCare Texas Fort Worth, TX United States

**Keywords:** artificial intelligence, AI, Delphi, disparities, disparity, engagement, equitable, equities, equity, ethic, ethical, ethics, fair, fairness, health disparities, health equity, humanitarian, machine learning, ML

## Abstract

**Background:**

Artificial intelligence (AI) and machine learning (ML) technology design and development continues to be rapid, despite major limitations in its current form as a practice and discipline to address all sociohumanitarian issues and complexities. From these limitations emerges an imperative to strengthen AI and ML literacy in underserved communities and build a more diverse AI and ML design and development workforce engaged in health research.

**Objective:**

AI and ML has the potential to account for and assess a variety of factors that contribute to health and disease and to improve prevention, diagnosis, and therapy. Here, we describe recent activities within the Artificial Intelligence/Machine Learning Consortium to Advance Health Equity and Researcher Diversity (AIM-AHEAD) Ethics and Equity Workgroup (EEWG) that led to the development of deliverables that will help put ethics and fairness at the forefront of AI and ML applications to build equity in biomedical research, education, and health care.

**Methods:**

The AIM-AHEAD EEWG was created in 2021 with 3 cochairs and 51 members in year 1 and 2 cochairs and ~40 members in year 2. Members in both years included AIM-AHEAD principal investigators, coinvestigators, leadership fellows, and research fellows. The EEWG used a modified Delphi approach using polling, ranking, and other exercises to facilitate discussions around tangible steps, key terms, and definitions needed to ensure that ethics and fairness are at the forefront of AI and ML applications to build equity in biomedical research, education, and health care.

**Results:**

The EEWG developed a set of ethics and equity principles, a glossary, and an interview guide. The ethics and equity principles comprise 5 core principles, each with subparts, which articulate best practices for working with stakeholders from historically and presently underrepresented communities. The glossary contains 12 terms and definitions, with particular emphasis on optimal development, refinement, and implementation of AI and ML in health equity research. To accompany the glossary, the EEWG developed a concept relationship diagram that describes the logical flow of and relationship between the definitional concepts. Lastly, the interview guide provides questions that can be used or adapted to garner stakeholder and community perspectives on the principles and glossary.

**Conclusions:**

Ongoing engagement is needed around our principles and glossary to identify and predict potential limitations in their uses in AI and ML research settings, especially for institutions with limited resources. This requires time, careful consideration, and honest discussions around what classifies an engagement incentive as meaningful to support and sustain their full engagement. By slowing down to meet historically and presently underresourced institutions and communities where they are and where they are capable of engaging and competing, there is higher potential to achieve needed diversity, ethics, and equity in AI and ML implementation in health research.

## Introduction

Recent events and academic literature have underscored a role for the field of artificial intelligence (AI) and machine learning (ML) technology to take all stakeholders’ impressions and concerns into account to inform approaches for achieving health equity [1-5]. It has also become imperative to strengthen AI and ML literacy in underserved communities and build a more diverse workforce in AI and ML design and development. However, whether as a practice or as an academic discipline, AI and ML are not yet engineered to address all sociohumanitarian issues and complexities. This is especially true for socially and economically marginalized communities whose members are frequently unheard or have limited engagement in research, discovery, and innovation pipelines for cultivating shared prosperity.

The general population still has limited knowledge about AI and ML, with 1 study reporting that only about one-quarter of people have heard of AI or ML, and only about half are at least somewhat aware of AI and ML [6]. Furthermore, individuals and communities who are subject to potentially detrimental outcomes (persons with mental health care needs and disabilities, persons with marginalized racial or ethnic identities, etc) may be more aware of the potential harms of AI and ML, particularly when it comes to the risk of harm from bias [7,8]. Thus, people who are presently or historically underserved or marginalized may be particularly concerned that they will be harmed by AI or ML technologies, especially in cases where AI or ML is used or applied without their awareness.

The overall lack of understanding about AI and ML and the awareness of bias among historically and presently marginalized populations could result in limited trust in the technology and its use. To build trust among those most subject to bias or at risk of detrimental outcomes, it is critical for AI and ML developers to assess their own reliability and adapt their practices to build trustworthiness with the most vulnerable stakeholders. In this context, it is also important to recognize that trust varies across and within populations, and people may have more or less trust in health care technologies based on factors such as previous experience of racial bias [9].

If implemented responsibly, AI and ML has the power to account for and assess a variety of factors that contribute to health and disease to improve prevention, diagnosis, and therapy. The ability to predict the risk of adverse health outcomes and identify high-risk patients for targeted preventive interventions offers tremendous potential to improve the health of individuals and medically underserved populations [10,11].

A great deal of AI and ML today is developed without meaningful engagement of individuals and communities, even when those individuals and communities have (knowingly or unknowingly) generated data used by AI and ML models. When there are proactive efforts to engage communities in AI and ML design, development, or application, various factors may negatively affect how people respond (Textbox 1). For instance, failure to educate about AI and ML and contextualize its impact on an individual and their community may bias individuals’ consent to contribute data to build such technologies and, subsequently, lead to biased outcomes in terms of who benefits from the technology’s development and application. Consequently, poor engagement can exacerbate inequities in the creation, development, and application of AI and ML.

**Factors that may engender inequitable access to artificial intelligence (AI) and machine learning (ML) or demotivate participation in AI and ML.**

**Factors that demotivate participation in AI and ML**

- Cultural norms or expectations that discourage the use of AI and ML technology
- Fear and reservations that the AI and ML tool may be used to cause harm
- The history of major AI and ML–developing institutions is not inclusive of all communities, thus defying communities’ trust
- The lack of access to high-performance infrastructure and resources needed to execute AI and ML models
- The lack of interest, excitement, or perception of “hype”
- Unaddressed confusion, misinformation, or disillusionment

**Factors that exacerbate inequitable access to the benefit of AI and ML**

- Asymmetric ability to extract value from AI and ML
- Insufficient access to the internet, data, and data services (ie, digital divide)
- Insufficient funding or economic opportunities
- There is an intractable disagreement and power imbalance between stakeholders about how AI and ML should be used or applied
- Lack of institutional leadership or commitment
- Limited experience, knowledge, and education
- Sociocultural factors affecting digital access and inclusion

The underengagement of communities in research, development, and use of AI and ML often reflects limited knowledge and crucial misunderstandings about AI and ML, including how it is used in health care settings to advance health-related innovations and solutions. Thus, stronger, more targeted, and more intentional engagement is required to help these groups identify and address real or potential harms associated with the problematic implementation of AI and ML in high-consequence settings. To address this challenge, the US National Institutes of Health’s Artificial Intelligence/Machine Learning Consortium to Advance Health Equity and Researcher Diversity (AIM-AHEAD) was established in 2021 with a mission to address factors that undermine achieving health equity through the design, use, and application of AI and ML, including the lack of the following:

- An adequately diverse workforce
- Adequate data and data infrastructure
- Adequate community engagement
- Adequate oversight, governance, and accountability
- Consensus that ethics can strengthen innovation

The tension between individual desires and population needs challenges ethics and equity in AI and ML settings. Thus, the Ethics and Equity Workgroup (EEWG) was formed within the AIM-AHEAD Consortium to ensure that ethics and fairness are at the forefront of AI and ML applications to build equity in biomedical research, education, and health care. Activities within the workgroup have included deliberations and discussions to develop and reach consensus on actionable guiding principles, a glossary of key terms, and other engagement tools to encourage greater attention to ethics and equity in AI and ML development. This study describes these activities with the intent to serve and inform the AIM-AHEAD community of stakeholders; external consortia, organizations, and communities that have goals similar to the AIM-AHEAD; and those interested in ethical and equitable AI and ML development and applications more broadly.

## Methods

### Workgroup Establishment

**Figure 1:**
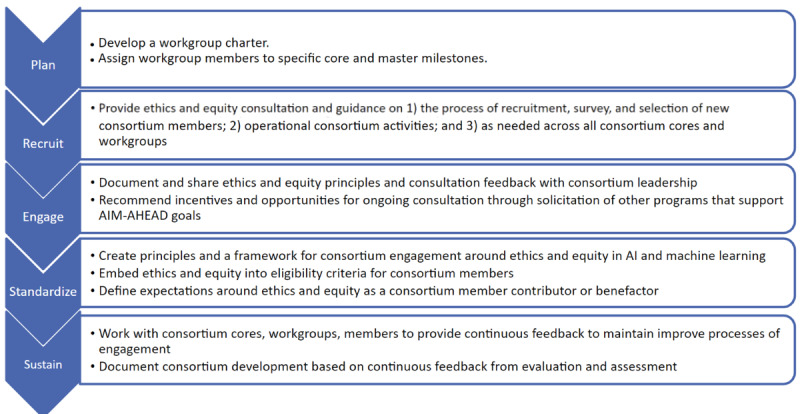
Artificial Intelligence/Machine Learning Consortium to Advance Health Equity and Researcher Diversity (AIM-AHEAD) Ethics and Equity Workgroup’s scope of activities. AI: artificial intelligence.

The AIM-AHEAD EEWG was created in 2021 to guide the ethical and equitable development and implementation of AI and ML tools and processes broadly within the AIM-AHEAD. Simultaneously, an Equitable Policy Development Workgroup was developed within the AIM-AHEAD Infrastructure Core. To ensure rapid and coordinated progress with respect to embedding ethics and equity into AIM-AHEAD activities, both within and outside of the Infrastructure Core, the EEWG’s efforts were harmonized and merged with the Infrastructure Core’s Equitable Policy Development Workgroup upon recommendation by the EEWG cochair and multiple principal investigators for the AIM-AHEAD Infrastructure Core. The newly reconfigured EEWG began by defining its scope of activities (Figure 1).

### Workgroup Membership

At the start of the program in year 1, the EEWG was comprised of 51 members (AIM-AHEAD principal investigators and coinvestigators) and 3 cochairs. AIM-AHEAD participants either requested to join or were selected to join by their project leaders within the program. During year 2, the EEWG’s membership was consolidated into 2 cochairs and approximately 40 AIM-AHEAD principal investigators, coinvestigators, leadership fellows, and research fellows. This reduction in EEWG cochairs and members occurred for two main reasons: (1) time and effort among members were reallocated to other activities within the AIM-AHEAD (administrative planning for regional hubs, research, etc), and (2) given the evolution of the program over time, the year 1 members were provided an opportunity to recommit to the EEWG for year 2. In both years, EEWG cochairs and members represented a variety of academic disciplines and focus areas, including but not limited to medicine, computational science, population health, health science, data science, bioethics, law, community engagement, human-centered design, health disparities research, biological science, social science, and engineering.

### Development of a Set of Ethical Principles for AI and ML

The initial effort of the EEWG during year 1 was to produce a set of principles and a glossary to inform the practice of ethics and equity in AI and ML development and implementation in health research. During year 1, members convened in weekly meetings that led to consensus on the development of specific workgroup deliverables. EEWG members reviewed the literature to identify relevant sources with perspectives on ethics, equity, and social determinants of health, especially those that were community driven, and lessons that could inform the development and use of AI and ML in health disparity and disease prevention research [12-27].

To develop the principles, the EEWG used a modified Delphi approach to facilitate discussions around tangible steps that the Consortium should take to ensure that ethics and fairness are at the forefront of AI and ML applications to build equity in biomedical research, education, and health care [28]. Specifically, the EEWG engaged in weekly (year 1) and biweekly (year 2) meetings to suggest, review, and deliberate a corpus of published content and literature considered useful toward integrating ethics and equity into AI and ML development and contributed original thought leadership and content in reaction to the content and literature reviewed to devise actionable principles. The EEWG approached the development of the principles with optimism about the potential of AI and ML to address health disparities by empowering communities, yet with recognition of complex societal challenges: inadequate or misrepresentation in data sets, algorithmic bias, imbalances in communities’ access to data and information about themselves, misuses of AI and ML tools, and threats to the civil and human rights of individuals and communities who are or may be subject to illegal or pervasive AI and ML surveillance, to name just a few.

### Development of a Glossary

To develop the glossary, during year 1, the EEWG began by defining ways in which outputs of AI and ML can (1) fail to be informative or useful for individuals and groups; (2) distinguish among individuals in inappropriate ways as a result of bias, failure of inclusion, or misuse; or (3) be poorly vetted by individuals and groups who are or may be subject to potentially harmful actions and decisions made by key or authoritative stakeholders that rely on AI and ML for decision support as a result of insufficient engagement with key stakeholders, including data participants.

Using a modified Delphi approach that likewise involved polling, ranking, and other exercises, consensus was reached on terms to define [29]. During its meetings, the EEWG discussed all possible terms that would be key to define to inform the ethical and equitable development and application of AI and ML, followed by 2 rounds of ranking and polling exercises to narrow their suggestions to 12 sentinel terms. Sentinel terms discussed during meetings, for example, included demographical terms such as self-defined or assigned race, ethnicity, sex, ability, and gender that can lead to errors in the development of AI and ML, which can in turn lead to potentially irreversible, intergenerational, and multigenerational harm to individuals and groups subjected to decisions informed by or based on AI and ML outputs. During year 2, remote meetings were held on a biweekly basis to further deliberate and refine the principles and glossary. Refinements were based on expert stakeholder feedback gathered through a survey among participants in the AIM-AHEAD pilot project and during remote convenings.

### Development of an Interview Guide

The EEWG initially sought to conduct a quantitative survey to assess how AIM-AHEAD researchers would implement the principles in practice. A draft survey was developed by 2 volunteers within the workgroup, who later shared the draft survey with the broader workgroup for iterative feedback and edits during weekly (year 1) and biweekly (year 2) meetings. The draft survey was also shared with awardees of AIM-AHEAD pilot projects for feedback. As the EEWG deliberated on the feedback, it ultimately determined that a qualitative interview (vs a quantitative survey) would be a more useful approach to garnering AIM-AHEAD researchers’ perspectives on implementing the principles in practice. Thereafter, the EEWG met regularly to convert the quantitative survey into an interview guide with the intent of learning the interviewees perspectives and natural reactions to the AIM-AHEAD ethics and equity principles and glossary.

### Ethical Considerations

The EEWG’s efforts in developing the interview guide and conducting the interviews were focused exclusively on program-specific planning for the AIM-AHEAD and were not intended as human subjects research. AIM-AHEAD investigators’ responses to the interviews were wholly voluntary, and their comments were used exclusively to develop the program’s principles and were subject to further assessment for generalizable knowledge.

## Results

### AIM-AHEAD Ethics and Equity Principles

#### Overview

Based on the EEWG’s internal Delphi process, informed by insights from interviews with AIM-AHEAD investigators, the workgroup articulated 5 core principles, each with subparts, which articulate best practices for working with stakeholders from historically and presently underrepresented communities.

1. Build trust with communities
2. Design and implement AI and ML with intention
3. Cocreate, do not dictate
4. Build capacity
5. Reset the rules

#### Build Trust With Communities

Researchers should build trust and share power to enable data-driven decision-making among multiple partners—this must be earned through longstanding, sustained relationships in the community, which takes time, investment, and resources to manifest.

- Through authentic community engagement, determine, understand, and deliver value in a manner that is community driven, community defined, and community led.
- Use asset-based language and thinking in collecting, interpreting, and reporting community-level data (in lieu of deficit-based language and thinking).
- Be transparent about the structure of AI models, data that are contextually limited or incomplete, and limitations in the capabilities of data analytics tools and platforms.
- Commit to ongoing engagement and bidirectional communication between AI and ML developers and communities around interventions to address limitations in the capabilities of data analytics tools and platforms.

#### Design and Implement AI and ML With Intention

Researchers should take collective action and engage in data-driven decision-making toward embedding equity, which requires shared goal setting, design, implementation, and accountability.

- Determine shared goals that serve as a commitment anchor and barometer for cocreated actions.
- Design with intent to overcome root causes of bias to solve or address (vs merely explore) an immediate, ongoing, or systemic problem affecting communities experiencing certain hardships that have contributed to health inequity.
- Develop and implement ongoing AI and ML design mechanisms and procedures to monitor AI and ML algorithms with the goal of preventing or mitigating harm.

#### Cocreate, Do Not Dictate

Researchers should move from superficial community engagement to true community partnership through meaningful cocreation.

- Develop AI and ML infrastructure, protocols, and programs in partnership with key and affected community stakeholders.
- Avoid tokenizing individuals and communities to achieve asymmetric goals that are or can be perceived as to the detriment of communities.
- Limit the use of computational methods that are or can be perceived as a substitution for data that would be only obtained through strong community engagement.
- Be transparent about the short-, medium-, and long-term sponsorships, investors in, and potential beneficiaries of AI and ML projects.

#### Build Capacity

Researchers should invest in people, data, and computational technology—today, as community leaders dig into this work, and tomorrow, as society collectively builds a stronger, more diverse tech talent pipeline.

- Educate stakeholders to enable AI and ML competency across clinical practice, community, and research settings (eg, build AI and ML model fact labels that can summarize or explain algorithms).
- Develop a plan to promote eHealth literacy in marginalized and underserved communities and groups.
- Build equitable access to AI and ML technology, its development, applications, and uses across real-world health contexts including social determinants of health and research.
- Develop a plan for building capacity that includes hiring and supporting a diverse workforce, dedicating funds for sustaining an existing workforce, and creating metrics that allow institutions to measure their success.

#### Reset the Rules

Researcher should reexamine the mechanisms that hold institutions accountable and resist the urgency of quick fixes to complex issues like systemic racism.

- Engage communities to determine their experiences with and desires to overcome the digital divide and facilitate the equitable inclusion and consideration of populations in AI and ML models and algorithms.
- Create equitable and liberated access to AI and ML development, implementation, and maintenance to oversee and correct model drift and guide entities in their reactions to AI and ML outputs.
- Identify and correct information asymmetries that may lead to communities’ lacking pertinent, actionable, and critical information that is exclusively held by powerful institutions.

### AIM-AHEAD Ethics and Equity Glossary Terms

Developers of AI and ML platforms and tools must contemplate, anticipate, mitigate, and address potential issues with downstream data aggregation, interpretation, and use. Meeting these goals requires a shared understanding of the terms used in these policies and processes. The EEWG determined that, in many cases, sensitive demographic characteristics (eg, race, ethnicity, sex, ability, and gender) are particularly problematic as variables used in AI and ML because they are often inappropriately understood as being rooted solely or primarily in genetic or phenotypic differences rather than strongly influenced by discriminatory sociohistorical and sociocultural practices.

To capture and promote a shared understanding of key terms, the EEWG developed a glossary of 12 words (Table 1) out of 28 considered that follow or build upon existing understandings of these concepts, highlighting their particular importance for the optimal development, refinement, and implementation of AI and ML.

**Figure 2:**
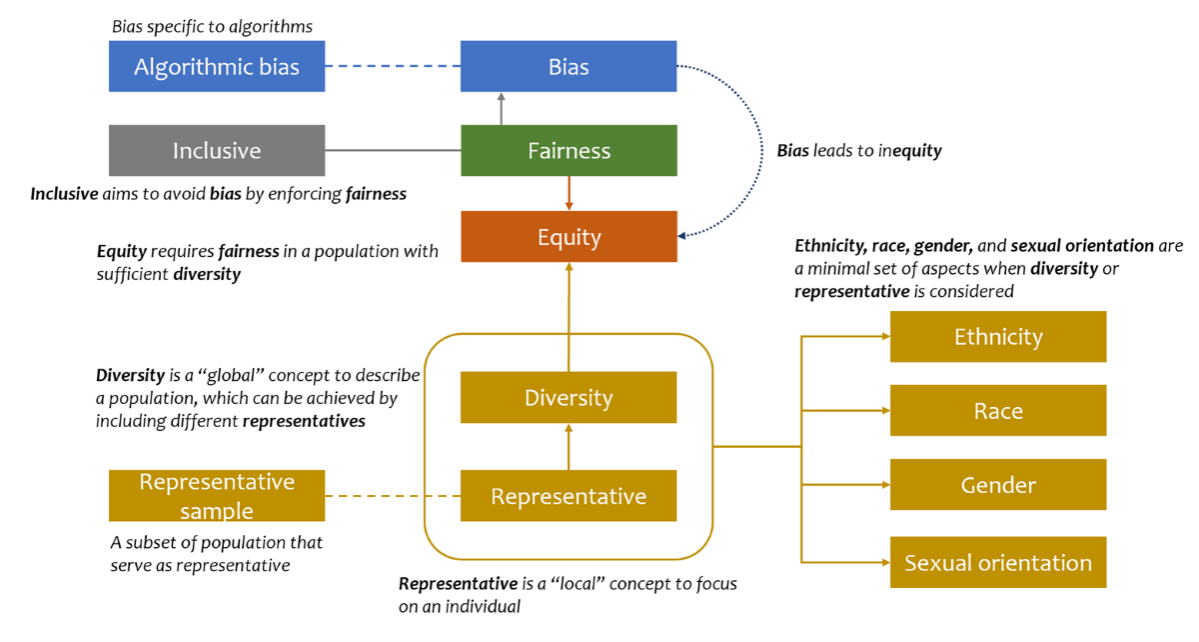
Definitional concepts of Artificial Intelligence/Machine Learning Consortium to Advance Health Equity and Researcher Diversity (AIM-AHEAD) ethics and equity glossary terms.

In addition, the EEWG developed a concept relationship diagram that describes the logical flow of and relationship between the definitional concepts described in Table 1 and Figure 2. The center of this diagram is equity, which requires AI developers and implementers to enforce fairness and avoid bias in a population with sufficient diversity by being inclusive. To implement diversity, representatives that are characterized by a minimal set of aspects—ethnicity, race, gender, and sexual orientation—need to be collected. They will form a representative sample if they can reflect the characteristics of a population. A representative sample can mitigate algorithmic bias, which is one specific type of bias.

**Table 1:** Artificial Intelligence/Machine Learning Consortium to Advance Health Equity and Researcher Diversity (AIM-AHEAD) ethics and equity glossary terms and definitions.

No	Glossary term	AIM-AHEAD definition
1	Ethnicity	Distinct patterns of language, lifestyle, illness, and health beliefs encountered among an individual or representative population, regardless of race, and that may subject the individual or population to bias or discrimination.
2	Race	A social construct or assumption based on patterns in an individual’s or representative population’s language, lifestyle, and health beliefs and immutable characteristics, such as skin tone, color, or hair texture, regardless of immigration status, socioeconomic status, genetic ancestry, or geographic origin, that may subject the individual’s or population to bias, structural racism, or discrimination that would warrant corrective antiracism actions.
3	Bias	Systematic error in information originating, gathering, or assessment activities, leading to selecting or encouraging one outcome or answer over others, which can result in human decisions and values that echo societal or historical inequities and produce inconclusive or limited assumptions about the broader population.
4	Equity	Equity is fairness and justice in policy, practice, and opportunity designed to address the distinct challenges of nondominant social groups with an eye to progressive outcomes. Health equity is the state in which everyone has the opportunity to attain full health potential, and no individual is disadvantaged from achieving this potential because of social position or any other socially defined circumstance.
5	Algorithmic bias	Systematic and repeated errors in the collection and consideration of a variety of factors, including but not limited to the design of the algorithm; unintended or unanticipated use or decisions relating to the way data are collected, represented, or used; lack of sensitivity to identity factors that contribute to bias in the evaluation of the algorithm, or misappropriation of the algorithm through miscommunicating or misunderstanding its limitations.
6	Diversity	The wide variety of shared and different personal and group characteristics among human beings. There are many kinds of diversity, including gender, sexual orientation, class, age, country of origin, education, religion, geography, physical or cognitive abilities, or other characteristics. Valuing diversity means recognizing differences between people, acknowledging that these differences are a valued asset, and striving for diverse representation as a critical step toward equity.
7	Inclusive	Avoiding bias by providing equitable and open access to opportunities and resources for engagement. This can be accomplished, for example, by enforcing fairness in the data collection methods, enforcing fairness in the assignment of labels, developing explainable, transparent, and interpretable models, having diverse teams monitor models, and looking for biases and eliminating them.
8	Fairness	Intent to promote nondiscrimination and population representation when assessing a group’s eligibility for a benefit or penalty. This is particularly important given the statistical likelihood that artificial intelligence and machine learning systems could produce discriminatory outputs once algorithms are implemented across one or more data sets.
9	Representative	An individual or body chosen or appointed to act or speak for an individual, population, or subpopulation sharing a set of features or characteristics, including but not limited to gender, race, or sexual orientation.
10	Representative sample	A subset of a population that reflects the characteristics of the entire population from which it has been selected.
11	Gender identity	An individual’s sense of oneself as male, female, or something else. When an individual’s gender identity and biological sex are not congruent, the individual may identify along the transgender spectrum. An individual may choose to change their gender one or more times. Varying cultural indicators of gender, such as clothing choice, speech patterns, and personality traits, relate to gender but are not acceptable means to determine another’s gender identity. The change in an individual’s gender can be used to abuse, discriminate against, and misrepresent individuals and groups.
12	Sexual orientation	An individual’s capacity for attraction to and sexual activity with the same or different sex. An individual’s sexual orientation is indicated by one or more of the following: how an individual identifies their own sexual orientation, an individual’s capacity for experiencing sexual and affectional attraction to people of the same or different gender, and an individual’s sexual behavior with people of the same or different gender. Sexual orientation incorporates three core ideas: consensual human relationships—sexual, romantic, or both—the biological sex of an individual’s actual or potential relationship partners, and enduring patterns of experience and behavior. Sexual minorities, or people whose sexual orientation does not conform to heteronormative cultural expectations, are vulnerable to violence and discrimination.

### Interview Guide

As mentioned, extensive and iterative feedback received during the development of the quantitative survey led the EEWG cochairs and members to determine that a qualitative engagement approach is warranted to facilitate meaningful and diverse stakeholder engagement to disseminate and facilitate implementation of the principles and glossary. Therefore, the EEWG developed an interview guide that can be used or adapted to garner and understand AIM-AHEAD members’ and other community perspectives on the principles and glossary. The interview guide is provided in Multimedia Appendix 1.

## Discussion

### Overview

The role of those who will be affected by the findings of the research enterprise has evolved from their initial role as objects, as illustrated in the iconic painting of Edward Jenner administering the life-saving inoculation of the English boy with cowpox in 1796, the multiepisode television documentary “Microbes and Men,” and the abuses of Black men in the US Public Health Service Study of the natural history of untreated syphilis at Tuskegee [30-33]. Over time, more attention has been devoted to assessing the potential harms and benefits of research to the people who are studied, albeit primarily as viewed by investigators, typically White men, and institutional review boards, typically comprised of researchers with minimal or latent community involvement. Incentivizing representation of nonscientific, nonaffiliate community members on institutional review boards, engaging members of historically underrepresented groups in more visible roles as investigators, and engaging minority-serving institutions as partners in AI and ML research is necessary to promote equitable access to opportunities and careers in AI and ML. Such an intentional approach also, importantly, demonstrates an appreciation for local knowledge and facilitates the design of more culturally informed interventions that consider how research will affect heterogeneous populations being studied in AI and ML research. This form of appreciation is necessary for tailoring engagement to the needs of diverse groups and understanding how to overcome barriers to AI and ML research and use [34].

Beyond promoting diverse and equitable opportunities for participation in AI and ML research, it is necessary to recognize the need to translate that work into actual practice, which historically has also been a barrier to health equity. For example, the association of the lower-quality data measured by pulse oximetry with dark skin tones has long been known, and there have been versions of the technology designed to account for this discrepancy, but versions of pulse oximeters with biased tendencies remain in wide use [35]. There is a real risk that AI and ML technology will follow a similar pathway if there is not sufficient action to build ethics and equity into the research.

Overall, our effort reported here achieves 2 goals. The first is to describe what is needed procedurally and substantively to achieve equity. This is a complex process that must take place and evolve over time. It cannot be addressed as a 1-time event or by filling out a checklist. Achieving equity requires rebalancing the interests at stake in research, which, at a minimum, means truly considering and addressing the interests of the people who will be affected by the results. Ideally, research participants can become cocreators as ethics in AI and ML and related ethical principles evolve into more commonly accepted policies and practices. The second goal of this reported effort is to emphasize that addressing equity requires an inclusive, ongoing process with a shared understanding of salient terms that will evolve over time. Recent engagements within the AIM-AHEAD program have noted this to be true even for terms like AI and ML, as today very few stakeholders have been able to clearly articulate how AI and ML can be or is used in the real world [34]. New and ongoing national initiatives, such as the National Academy of Medicine’s AI Code of Conduct project, which intends to develop a “code of conduct for the development and use of AI in health, medical care, and health research,” are encouraged to learn from the EEWG’s efforts [36].

Our work builds on and can be incorporated into current AI and ML ethics and equity frameworks and policies within and outside of the United States, focused on improving population health through broad community involvement in AI and ML application development [17,36-38]. This includes, but is not limited to, the National Institutes of Health’s policies and programs on AI and ML application development in health research; policy developments undertaken by the US Senate Health, Education, Labor, and Pensions Committee; the National Academy of Medicine’s Artificial Intelligence Code of Conduct project; the European Commission’s Guidelines for Trustworthy AI; Asilomar AI Principles; and lastly and importantly, a groundbreaking and recent US White House Executive Order explicitly supporting the mission of the AIM-AHEAD [36,39-42].

Importantly, our work provides a complementary, fundamental, and basic blueprint or process, along with operational tools and building blocks, to educate stakeholders on this practice of creating safe spaces and setting culture tones for diverse stakeholder engagement and consensus around best practices and shared terminology. Also importantly, our tools enable the collection of ongoing and iterative feedback concerning the local implementation of our principles and glossary. Iterations may be further disseminated, along with public-facing endorsements of the principles and glossary in their current form, by like-minded stakeholders seeking to ensure that researcher diversity, community, and social justice concerns influence AI and ML application development processes in health research and, broadly, science and technology.

Inclusive and ongoing processes to develop a shared understanding of salient terms like AI and ML and those described in our glossary require more time, greater inclusion, and deeper incorporation of diverse community perspectives. This approach differs drastically from the typical project life cycles afforded by the gold rush mentality that has emerged with AI and ML today. Therefore, one key step, moving forward, would be to persuade leaders in the AI and ML research enterprise to broadly disseminate the lessons that may be learned in operationalizing our EEWG principles and glossary. Programs such as the AIM-AHEAD need to objectively assess their administrative processes and evaluation criteria for what constitutes ethical and equitable opportunities for an AI and ML investigation, including investigator inclusion, data governance, data sources, and data infrastructure.

There are limitations to consider in our process and recommendations. First, the EEWG has continuously revisited the principles and glossary for potential editing based on the members’ evolving experience and expert opinions, even though making these deliverables “living documents” complicates the process of achieving sustainable consensus. Nonetheless, the principles and glossary will require reflection, appreciation, and adjustments over time to account for the effects of real-world events, human choices, or interpersonal phenomena from relevant perspectives. Also, some of our proposed glossary terms may already be limited in scope with respect to real-world events and phenomena. For instance, although our definition of “representative” concerns “an individual or body chosen or appointed to act or speak for an individual, population, or subpopulation,” there are certain matters in which a representative may be self-appointed without specific authorization from those they wish to represent.

Therefore, ongoing engagement around the use of our principles and glossary in AI and ML research settings is encouraged to maximize their potential benefits and minimize any potential harm. However, ongoing engagement with institutions that have limited resources to support their full participation requires careful consideration and discussion of how to incentivize, support, and sustain meaningful engagement beyond mere compensation. One way to accomplish this is to seek institutional input through authentic connections to determine what they consider a valuable investment for their time, instead of deciding for them. For example, such connections can be made both within and outside of conferences, convenings, and events hosted by minority-serving institutions nationwide (eg, the Annual Biomedical Research Conference for Minoritized Scientists or the National Society of Black Engineers’ Annual Convention).

### Conclusions and Next Steps

An overemphasis on speed or velocity works against taking the time needed to foster the inclusion of historically and presently underrepresented communities in the development of AI and ML, ultimately rewarding AI and ML “haves” over “have-nots.” In the private sector (eg, big technology companies and startups), the pace of AI and ML development is extremely rapid and difficult to manage. Inequitable divisions in access to resources like computers, smartphones, and the internet have vastly decreased over the past decade. Yet, AI and ML technology that is used with adequate operational know-how and e-literacy, cost of use, human resources and staffing needs to maintain cyberinfrastructure, and many other technical and nontechnical resources, is where these inequitable divisions can be addressed.

An equity-oriented public sector intervention, such as the AIM-AHEAD, can be more effective in achieving diversity and inclusion goals by emphasizing actions that do not sacrifice trust-building for the sake of rapid development of technology, especially in the initial stages. By slowing down to meet historically and presently underresourced institutions and communities where they are and where they are capable of engaging and competing, we can more effectively evaluate AI and ML implementation and results for bias over time and expand the potential to achieve the aims of ethics and equity. We envision a virtuous cycle of shared learning, building on our EEWG deliverables, that may bridge researchers and impacted communities into a new intersection of computational sciences, ethics, and health equity.

## Appendix

Multimedia Appendix 1 Interview guide.

## Abbreviations

AI: artificial intelligence
AIM-AHEAD: Artificial Intelligence/Machine Learning Consortium to Advance Health Equity and Researcher Diversity
EEWG: Ethics and Equity Workgroup
ML: machine learning
