# Supplementary material for: Developing Ethics and Equity Principles, Terms, and Engagement Tools to Advance Health Equity and Researcher Diversity in AI and Machine Learning: Modified Delphi Approach
Source: JMIR AI. 2023 Dec 6;2:e52888. doi: 10.2196/52888 (PMC11041493; doi:10.2196/52888)
Supplement: Multimedia Appendix 1 [file ai_v2i1e52888_app1.docx]

**Appendix 1**. Interview Guide to Facilitate Diverse Stakeholder Engagement on AIM-AHEAD Ethics and Equity Principles and Glossary

**Agenda:**

- Introductions from Project Team
- Discuss the purpose and goals for the interview (to learn the interviewees perspectives around and natural reactions to the AIM-AHEAD Ethics and Equity Principles and Glossary).
  - Note that the interview should take no more than one hour of their time.
- Ask for Permission to Record
- Encourage the interviewer to pursue threads in the conversation as they arise, and ask follow up questions to flesh out details.
- Request Introduction from Interviewee including name, organization, role in AIM AHEAD
- Please take a moment to review the AIM-AHEAD Ethics and Equity Principles.
  - Provide up to 15 minutes at the start of the interview to help familiarize the interviewee with the Principles and Glossary
- Can you describe to us which principle resonates with you the most?
  - If any, ask the participant to indicate which principle(s) and discuss why the principle(s) resonates with the most.
- Can you describe which principle(s) applies the most to your work?
  - If any, ask the participant to indicate which principle(s) and discuss why the principle(s) applies to their work the most.
- Can you describe which principle(s) applies the least to your work?
  - If any, ask the participant to indicate which principle(s) and discuss why the principle(s) applies to their work the least.
- Would you like to share any experiences within your scope of work or interests that relate to one or more of these principles?
  - Based on those experiences alone, what are your natural reactions to principle(s)?
- Are there other important principles missing from this list?
  - If yes, ask the participant to elaborate.
- Looking at the AIM-AHEAD Ethics and Equity Glossary, does any particular term stand out to you?
  - If no/yes, then ask the participant to elaborate.
- Do any of these terms align with your understanding of how they are or can be used within AI/ML?
- Would you like to share any additional thoughts, perspectives, reactions, and/or feedback concerning the AIM-AHEAD Ethics and Equity Glossary?
- On a scale of 1 to 5, how might you perceive the level of difficulty in implementing these principles within your institution?
  - very easy (1), somewhat easy (2), neutral (3), somewhat hard (4), very hard (5)
  - If examples are needed:
    - Applying any of these principles within existing institutional structures
    - Adhering to policies that may align or conflict with these principles
    - Changing existing policies and/or culture to apply these principles
- Can you describe one way in which you apply these principles in your work/research/institution?
- On a scale of 1 to 5, how valuable are these principles as they would pertain to your work and involvement within AIM-AHEAD?
  - not valuable (1), somewhat valuable (2), neutral (3), somewhat valuable (4), extremely valuable (5)
- On a scale of 1 to 5, how much value does this principals document bring to the field?
- When thinking of the principles, what three words come to mind?
- Is there anything you would like to share with us today before we conclude the interview?
- Closeout / Next Steps (will convene the full group to share and discuss findings during an EEWG meeting ahead of paper dissemination)
